# 1-(Benzotriazol-1-yl)-2-bromoethanone

**DOI:** 10.1107/S1600536812042900

**Published:** 2012-10-20

**Authors:** Abdullah M. Asiri, Nader E. Abo-Dya, Muhammad Nadeem Arshad, Muhammad Shafiq

**Affiliations:** a Chemistry Department, Faculty of Science, King Abdulaziz University, PO Box 80203, Jeddah 21589, Saudi Arabia; b Center of Excellence for Advanced Materials Research (CEAMR), Faculty of Science, King Abdulaziz University, PO Box 80203, Jeddah 21589, Saudi Arabia; c Department of Pharmaceutical Organic Chemistry, Faculty of Pharmacy, Zagazig University, Zagazig, 44519, Egypt; d Department of Chemistry, Government College University, Faisalabad 38040, Pakistan

## Abstract

In the title compound C_8_H_6_BrN_3_O, the benzotriazole ring is essentially planar (r.m.s. deviation = 0.0034 Å) and the bromo­acetyl unit is twisted at a dihedral angle of 15.24 (16)° with respect to it. In the crystal, pairs of C—H⋯O hydrogen bondings result in the formation of inversion dimers, forming *R*_2_^2^(12) rings, which are connected by further C—H⋯O inter­actions into chains extending along the *b-*axis direction.

## Related literature

For the biological activity of the title compound, see: Nakagawa *et al.* (1973 ▶). For the crystal structure of a closely related compound, see: Selvarathy Grace *et al.* (2012 ▶). For graph-set notation, see: Bernstein *et al.* (1995 ▶).
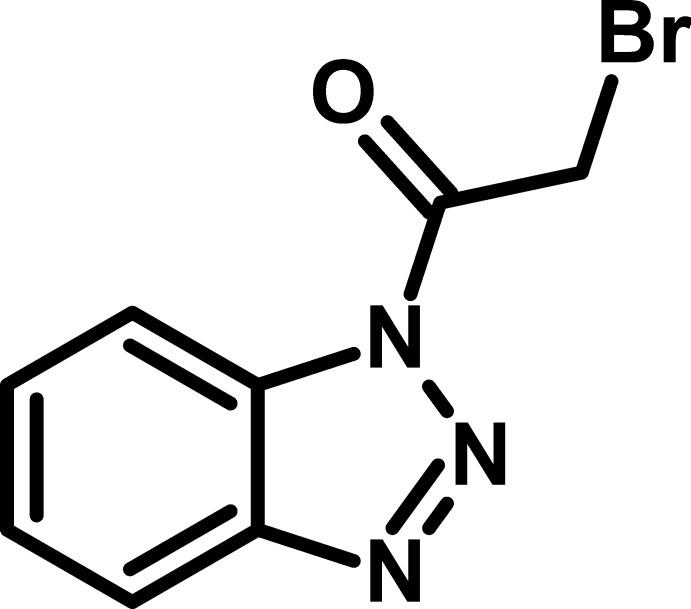


## Experimental

### 

#### Crystal data

C_8_H_6_BrN_3_O
*M* *_r_* = 240.07
Monoclinic, 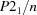

*a* = 12.4815 (4) Å
*b* = 4.7207 (1) Å
*c* = 15.4780 (5) Å
β = 103.468 (3)°
*V* = 886.91 (4) Å^3^
*Z* = 4
Cu *K*α radiation
μ = 6.02 mm^−1^
*T* = 296 K
0.28 × 0.11 × 0.05 mm

#### Data collection

Agilent SuperNova Dual (Cu at zero) Atlas, CCD diffractometer
Absorption correction: multi-scan (*CrysAlis PRO*; Agilent, 2012 ▶) *T*_min_ = 0.284, *T*_max_ = 0.753
4362 measured reflections
1833 independent reflections
1507 reflections with *I* > 2σ(*I*)
*R*_int_ = 0.034

#### Refinement

*R*[*F* ^2^ > 2σ(*F* ^2^)] = 0.044
*wR*(*F* ^2^) = 0.141
*S* = 1.07
1833 reflections
118 parameters
H-atom parameters constrained
Δρ_max_ = 0.43 e Å^−3^
Δρ_min_ = −0.51 e Å^−3^

### 

Data collection: *CrysAlis PRO* (Agilent, 2012 ▶); cell refinement: *CrysAlis PRO*; data reduction: *CrysAlis PRO*; program(s) used to solve structure: *SHELXS97* (Sheldrick, 2008 ▶); program(s) used to refine structure: *SHELXL97* (Sheldrick, 2008 ▶); molecular graphics: *PLATON* (Spek, 2009 ▶); software used to prepare material for publication: *WinGX* (Farrugia, 1999 ▶) and *X-SEED* (Barbour, 2001 ▶).

## Supplementary Material

Crystal structure: contains datablock(s) I, global. DOI: 10.1107/S1600536812042900/pv2595sup1.cif

Structure factors: contains datablock(s) I. DOI: 10.1107/S1600536812042900/pv2595Isup2.hkl

Supplementary material file. DOI: 10.1107/S1600536812042900/pv2595Isup3.cml

Additional supplementary materials:  crystallographic information; 3D view; checkCIF report

## Figures and Tables

**Table 1:** Hydrogen-bond geometry (Å, °)

*D*—H⋯*A*	*D*—H	H⋯*A*	*D*⋯*A*	*D*—H⋯*A*
C5—H5⋯O1^i^	0.93	2.50	3.266 (3)	139
C8—H8*B*⋯O1^ii^	0.97	2.47	3.413 (4)	163

Symmetry codes: (i) 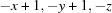; (ii) 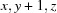.

**Fig. 1.**
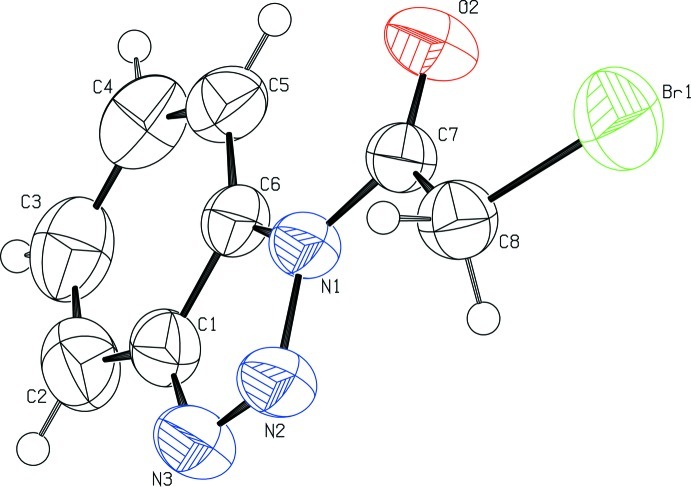
The molecular structure of the title compound with the atom numbering scheme. Displacement ellipsoids are drawn at the 50% probability level. H atoms are presented as small spheres of arbitrary radius.

**Fig. 2.**
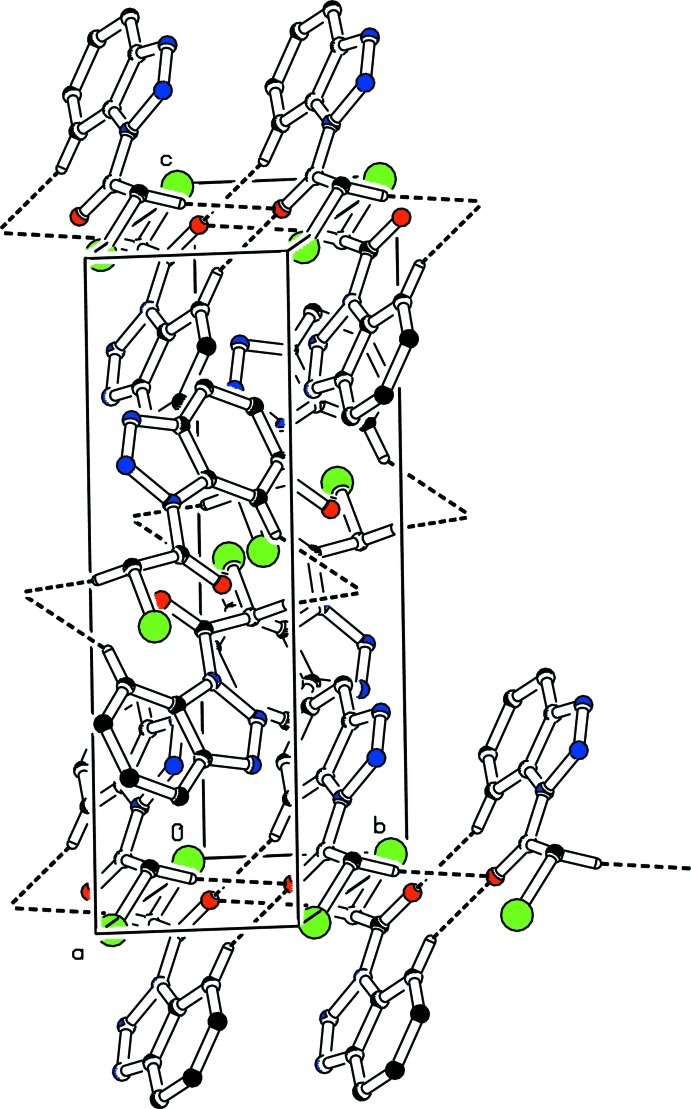
A view of the C—-H···O hydrogen bonds (dotted lines) in the crystal structure of the title compound. H atoms non-participating in hydrogen- bonding were omitted for clarity.

## supplementary crystallographic information

### Comment

The title compound shows antitumor activity mediated by inhibition of ascites sarcoma 180 in mice (Nakagawa *et al.*, 1973). Herein we report the crystal structure of the title compound.

The molecule of the title compound (Fig. 1) excluding Br1 atom is planer with r.m.s. deviation = 0.0367 Å; the Br1 atom is displaced from this plane by 0.489 (2) Å . The benzotriazole ring (C1–C6/N1–N3) is essentially planar with r.m.s.d = 0.0034 Å. The bromoacetyl moiety (Br1/C8/C7/O1) is twisted at a dihedral angle of 15.24 (16)° with respect to the benzotriazole ring. The structure of the title compound is stabilized by intermolecular hydrogen bonding interactions C5—H5···O1 resulting in dimers about inversion centers forming twelve membered ring motif *R*_2_^2^(12) (Bernstein *et al.*, 1995). The dimers are further connected through C8—H8B···O1 hydrogen bonding interactions resulting in chains of molecules extended along the *b* axis (Table 1 and Fig. 2).

### Experimental

A solution of thionyl chloride (0.4 mL, 5.5 mmol) and benzotriazole (1.79 g., 15 mmol) in methylene chloride (30 mL) was stirred at 293 K for 30 minutes. Bromoacetic acid (0.7 g., 5 mmol) was then added and the heterogeneous mixture was stirred for 2 hr. The solid was filtered and methylene chloride (50 mL) was added to the filtrate. The organic layer was extracted with saturated 4N HCl (3x, 15 mL), brine (2x, 5 mL) and dried over anhydrous Na_2_SO_4_. The crystals of the title compound suitable for X-ray crystallographic analysis were obtained by slow evaporation of methylene chloride (1.0 g, 83% yield).

### Refinement

The H-atoms were positioned with idealized geometry with C—H = 0.93 and 0.97 Å for aromatic and methylene H-atoms, respectively, and were refined as riding with *U*_iso_(H) = 1.2*U*_eq_(C).

Three reflections (5 2 15), (6 2 14) & (4 2 16) have been omitted in final refinement.

### Crystal data

**Table:**

C_8_H_6_BrN_3_O	*F*(000) = 472
*M**_r_* = 240.07	*D*_x_ = 1.798 Mg m^−^^3^
Monoclinic, *P*2_1_/*n*	Cu *K*α radiation, λ = 1.54184 Å
Hall symbol: -P 2yn	Cell parameters from 2295 reflections
*a* = 12.4815 (4) Å	θ = 3.6–76.0°
*b* = 4.7207 (1) Å	µ = 6.02 mm^−^^1^
*c* = 15.4780 (5) Å	*T* = 296 K
β = 103.468 (3)°	Plate, colorless
*V* = 886.91 (4) Å^3^	0.28 × 0.11 × 0.05 mm
*Z* = 4	

### Data collection

**Table:**

Agilent SuperNova Dual (Cu at zero) Atlas, CCD diffractometer	1833 independent reflections
Radiation source: SuperNova (Cu) X-ray Source	1507 reflections with *I* > 2σ(*I*)
Mirror monochromator	*R*_int_ = 0.034
ω scans	θ_max_ = 76.2°, θ_min_ = 4.1°
Absorption correction: multi-scan (*CrysAlis PRO*; Agilent, 2012)	*h* = −15→12
*T*_min_ = 0.284, *T*_max_ = 0.753	*k* = −5→5
4362 measured reflections	*l* = −19→19

### Refinement

**Table:**

Refinement on *F*^2^	Primary atom site location: structure-invariant direct methods
Least-squares matrix: full	Secondary atom site location: difference Fourier map
*R*[*F*^2^ > 2σ(*F*^2^)] = 0.044	Hydrogen site location: inferred from neighbouring sites
*wR*(*F*^2^) = 0.141	H-atom parameters constrained
*S* = 1.07	*w* = 1/[σ^2^(*F*_o_^2^) + (0.0838*P*)^2^ + 0.0945*P*] where *P* = (*F*_o_^2^ + 2*F*_c_^2^)/3
1833 reflections	(Δ/σ)_max_ < 0.001
118 parameters	Δρ_max_ = 0.43 e Å^−^^3^
0 restraints	Δρ_min_ = −0.51 e Å^−^^3^

### Special details

**Table:**

Geometry. All esds (except the esd in the dihedral angle between two l.s. planes) are estimated using the full covariance matrix. The cell esds are taken into account individually in the estimation of esds in distances, angles and torsion angles; correlations between esds in cell parameters are only used when they are defined by crystal symmetry. An approximate (isotropic) treatment of cell esds is used for estimating esds involving l.s. planes.
Refinement. Refinement of F^2^ against ALL reflections. The weighted R-factor wR and goodness of fit S are based on F^2^, conventional R-factors R are based on F, with F set to zero for negative F^2^. The threshold expression of F^2^ > 2sigma(F^2^) is used only for calculating R-factors(gt) etc. and is not relevant to the choice of reflections for refinement. R-factors based on F^2^ are statistically about twice as large as those based on F, and R- factors based on ALL data will be even larger.

### Fractional atomic coordinates and isotropic or equivalent isotropic displacement parameters (Å^2^)

**Table:**

	*x*	*y*	*z*	*U*_iso_*/*U*_eq_	
Br1	0.84802 (3)	0.99732 (9)	−0.01180 (3)	0.0947 (2)	
O1	0.63342 (16)	0.7775 (4)	0.02369 (12)	0.0717 (5)	
N1	0.62518 (17)	1.0238 (4)	0.14622 (14)	0.0512 (5)	
N2	0.67298 (17)	1.2209 (5)	0.20998 (14)	0.0603 (5)	
N3	0.61989 (19)	1.2185 (5)	0.27134 (14)	0.0673 (6)	
C1	0.5348 (2)	1.0227 (5)	0.25097 (18)	0.0582 (6)	
C2	0.4553 (3)	0.9458 (7)	0.2965 (2)	0.0774 (8)	
H2	0.4528	1.0294	0.3504	0.093*	
C3	0.3809 (2)	0.7419 (8)	0.2586 (2)	0.0800 (9)	
H3	0.3269	0.6862	0.2875	0.096*	
C4	0.3843 (2)	0.6154 (7)	0.1776 (2)	0.0743 (7)	
H4	0.3320	0.4787	0.1540	0.089*	
C5	0.4624 (2)	0.6864 (5)	0.13167 (17)	0.0608 (6)	
H5	0.4647	0.6012	0.0780	0.073*	
C6	0.53792 (18)	0.8945 (5)	0.17058 (14)	0.0509 (5)	
C7	0.6721 (2)	0.9628 (5)	0.07435 (16)	0.0518 (5)	
C8	0.7682 (2)	1.1462 (6)	0.06834 (17)	0.0614 (6)	
H8A	0.8172	1.1633	0.1268	0.074*	
H8B	0.7419	1.3343	0.0489	0.074*	

### Atomic displacement parameters (Å^2^)

**Table:**

	*U*^11^	*U*^22^	*U*^33^	*U*^12^	*U*^13^	*U*^23^
Br1	0.0844 (3)	0.1209 (5)	0.0895 (4)	0.00843 (18)	0.0419 (2)	−0.00526 (18)
O1	0.0855 (12)	0.0685 (12)	0.0622 (10)	−0.0108 (9)	0.0193 (9)	−0.0213 (9)
N1	0.0541 (10)	0.0483 (10)	0.0484 (10)	0.0024 (7)	0.0065 (8)	−0.0066 (7)
N2	0.0655 (11)	0.0525 (11)	0.0594 (11)	0.0003 (9)	0.0075 (9)	−0.0128 (9)
N3	0.0805 (13)	0.0640 (13)	0.0562 (11)	0.0053 (11)	0.0136 (10)	−0.0146 (9)
C1	0.0637 (14)	0.0573 (14)	0.0522 (13)	0.0132 (10)	0.0110 (11)	−0.0013 (9)
C2	0.087 (2)	0.0816 (19)	0.0684 (18)	0.0213 (16)	0.0281 (15)	0.0064 (14)
C3	0.0694 (15)	0.087 (2)	0.088 (2)	0.0074 (15)	0.0282 (15)	0.0204 (17)
C4	0.0606 (14)	0.0732 (18)	0.0864 (19)	−0.0028 (13)	0.0115 (13)	0.0139 (16)
C5	0.0592 (12)	0.0586 (14)	0.0600 (13)	−0.0012 (11)	0.0045 (10)	0.0011 (11)
C6	0.0535 (11)	0.0480 (11)	0.0486 (11)	0.0085 (9)	0.0066 (8)	0.0032 (9)
C7	0.0564 (12)	0.0495 (12)	0.0477 (12)	0.0042 (9)	0.0080 (9)	−0.0026 (8)
C8	0.0640 (13)	0.0619 (15)	0.0586 (13)	0.0006 (11)	0.0150 (10)	−0.0016 (11)

### Geometric parameters (Å, º)

**Table:**

Br1—C8	1.897 (3)	C2—H2	0.9300
O1—C7	1.199 (3)	C3—C4	1.398 (5)
N1—C6	1.376 (3)	C3—H3	0.9300
N1—N2	1.386 (3)	C4—C5	1.374 (4)
N1—C7	1.402 (3)	C4—H4	0.9300
N2—N3	1.279 (3)	C5—C6	1.397 (3)
N3—C1	1.388 (4)	C5—H5	0.9300
C1—C2	1.391 (5)	C7—C8	1.500 (4)
C1—C6	1.392 (3)	C8—H8A	0.9700
C2—C3	1.371 (5)	C8—H8B	0.9700
			
C6—N1—N2	109.9 (2)	C3—C4—H4	118.9
C6—N1—C7	129.1 (2)	C4—C5—C6	115.9 (3)
N2—N1—C7	120.8 (2)	C4—C5—H5	122.0
N3—N2—N1	108.2 (2)	C6—C5—H5	122.0
N2—N3—C1	109.8 (2)	N1—C6—C1	104.0 (2)
N3—C1—C2	131.0 (3)	N1—C6—C5	133.8 (2)
N3—C1—C6	108.2 (2)	C1—C6—C5	122.3 (2)
C2—C1—C6	120.7 (3)	O1—C7—N1	119.3 (2)
C3—C2—C1	117.3 (3)	O1—C7—C8	125.9 (2)
C3—C2—H2	121.4	N1—C7—C8	114.8 (2)
C1—C2—H2	121.4	C7—C8—Br1	112.09 (18)
C2—C3—C4	121.6 (3)	C7—C8—H8A	109.2
C2—C3—H3	119.2	Br1—C8—H8A	109.2
C4—C3—H3	119.2	C7—C8—H8B	109.2
C5—C4—C3	122.2 (3)	Br1—C8—H8B	109.2
C5—C4—H4	118.9	H8A—C8—H8B	107.9
			
C6—N1—N2—N3	0.0 (3)	C7—N1—C6—C5	−6.4 (4)
C7—N1—N2—N3	−174.6 (2)	N3—C1—C6—N1	−0.6 (3)
N1—N2—N3—C1	−0.4 (3)	C2—C1—C6—N1	179.7 (2)
N2—N3—C1—C2	−179.7 (3)	N3—C1—C6—C5	−179.9 (2)
N2—N3—C1—C6	0.6 (3)	C2—C1—C6—C5	0.4 (4)
N3—C1—C2—C3	180.0 (3)	C4—C5—C6—N1	−179.1 (2)
C6—C1—C2—C3	−0.4 (4)	C4—C5—C6—C1	0.0 (3)
C1—C2—C3—C4	0.0 (5)	C6—N1—C7—O1	1.4 (4)
C2—C3—C4—C5	0.4 (5)	N2—N1—C7—O1	174.8 (2)
C3—C4—C5—C6	−0.4 (4)	C6—N1—C7—C8	−179.3 (2)
N2—N1—C6—C1	0.4 (2)	N2—N1—C7—C8	−5.9 (3)
C7—N1—C6—C1	174.4 (2)	O1—C7—C8—Br1	−14.7 (3)
N2—N1—C6—C5	179.6 (2)	N1—C7—C8—Br1	166.13 (16)

### Hydrogen-bond geometry (Å, º)

**Table:**

*D*—H···*A*	*D*—H	H···*A*	*D*···*A*	*D*—H···*A*
C5—H5···O1^i^	0.93	2.50	3.266 (3)	139
C8—H8*B*···O1^ii^	0.97	2.47	3.413 (4)	163

Symmetry codes: (i) −*x*+1, −*y*+1, −*z*; (ii) *x*, *y*+1, *z*.
